# Thiolated Chitosan as an Intestinal Absorption Carrier with Hesperidin Encapsulation for Obesity Treatment

**DOI:** 10.3390/nu13124405

**Published:** 2021-12-09

**Authors:** Tzu-Chien Chen, Yu-Yu Ho, Rui-Chian Tang, Yong-Chen Ke, Jhih-Ni Lin, I-Hsuan Yang, Feng-Huei Lin

**Affiliations:** 1 Department of Biomedical Engineering, College of Medicine and College of Engineering, National Taiwan University, No. 49, Fanglan Rd., Taipei 10672, Taiwan; d07528022@ntu.edu.tw (T.-C.C.); paulho56@gmail.com (Y.-Y.H.); lala851206@gmail.com (Y.-C.K.); febe200630@gmail.com (J.-N.L.); tony910028@gmail.com (I.-H.Y.); 2 Department of Biochemical Science and Technology, College of Life Science, National Taiwan University, No. 1, Sec. 4, Roosevelt Rd., Taipei 10617, Taiwan; b06b02006@ntu.edu.tw; 3 Institute of Biomedical Engineering and Nanomedicine, National Health Research Institutes, No. 35, Keyan Rd., Zhunan, Miaoli County 35053, Taiwan

**Keywords:** obesity, chitosan, thiol, hesperidin

## Abstract

Obesity is characterized as abnormal or excessive fat accumulation harmful to one’s health, linked to hormonal imbalances, cardiovascular illness, and coronary artery disease. Since the disease stems mainly from overconsumption, studies have aimed to control intestinal absorption as a route for treatment. In this study, chitosan-thioglycolic acid (CT) was developed as a physical barrier in the gastrointestinal tracts to inhibit nutrient uptake. CT exhibits a superior mucoadhesive property compared to chitosan both in vitro and in vivo for the ability to form disulfide bonds with the intestinal mucosa. For CT as a potential drug delivery platform, hesperidin, a herb for bodyweight control in traditional Chinese medication, is encapsulated in CT and can be released consistently from this absorption barrier. In animal studies, CT encapsulated with hesperidin (CTH) not only results in a weight-controlling effect but limits adipose accumulation by hindering absorption, suggesting a potential role in obesity treatment. Neither CT nor CTH exhibit cytotoxicity or produce adverse immunological reactions in vivo.

## 1. Introduction

According to the WHO definitions, obesity and overweight refer to the excessive or abnormal fat accumulation that affects health [1]. The American Medical Association (AMA) officially defined obesity as a disease characterized by psychological symptoms, hormonal imbalance, and lack of neurotransmitters [2]. In 2014, there were more than 1.9 billion overweight adults, of whom 600 million were obese. It is estimated that 60% of adult males worldwide will be overweight or obese in 2030 [3], and the underlying childhood obesity problem is becoming gradually serious [4]. The problem of obesity is one of the most serious public health issues of the 21st century.

The etiology of obesity is caused by an energy imbalance. Energy imbalance is mainly caused by genes, diet, lifestyle, diseases, and drugs [5]. As well as congenital genetic defects, obesity may also be caused by Prader Willi syndrome or Alstrom syndrome [6,7]. Besides diseases such as Cushing’s syndrome and hypothyroidism, drugs such as steroids and insulin can also cause obesity [8,9].

Accordingly, studies have aimed to control intestinal absorption as a route of treatment, in which bariatric surgeries play a major role. There are two types of bariatric surgery, namely invasive and semi-invasive. The invasive methods include sleeve gastrectomy (SG), which changes the structure of the stomach to limit a patient’s appetite [10]; Furthermore, Roux-en-Y Gastric Bypass (RYGB) creates bypasses of the duodenum to prevent excess nutrients from being absorbed [11]. Nevertheless, these invasive surgeries irreversibly alter the gastrointestinal tracts, leaving only a small number of patients willing to undergo the surgeries. A semi-invasive method, EndoBarrier, utilizes an endoscopically delivered polymer film to block the absorption of nutrients to achieve weight control for patients with severe type 2 diabetes or morbidly obese patients. However, Endobarrier may cause abnormal proliferation where the device is installed. Additionally, it is required to be removed annually by endoscopy in a second operation, which may be uncomfortable for the patient [12,13,14]. As a result, this experiment aims to develop a polymer that can inhibit nutrient absorption in the small intestine to achieve bodyweight control.

Chitosan, a natural polysaccharide, has been studied for almost two decades in biomedical research because of its biodegradation, biodistribution, and low toxicity [15]. Additionally, the amino group on chitosan gives the material the ability to be easily modified [16,17]. Despite having been evaluated in a number of trials, the effectiveness of chitosan as a dietary supplement to reduce bodyweight remains in dispute [18]. Therefore, we used chitosan as the potential material to create a new type of absorption barrier material that is both safe and effective. The thiol group immobilizing strategy was selected for chitosan modification, for we hypothesized that the spontaneous disulfide bonds formed between thiomers and mucin on the intestine would enable improved adhesion. Moreover, it has been shown that the disulfide bonds formed by thiomers and mucin can be reversed [19,20]. As a consequence, the material may not be fixed permanently to the small intestinal mucosa.

The 1,4-butanediol diglycidyl ether (BDDE) was used in this study to crosslink chitosan with thioglycolic acid to synthesize chitosan-TGA (CT). To test the potential of CT to become a mucoadhesive drug-delivery platform, hesperidin was encapsulated in the CT as CTH. Hesperidin is a traditional Chinese medicinal material extracted from tangerine peel. It can reduce cholesterol, low-density lipoprotein, and triglycerides as shown in previous studies [21,22]. Therefore, we expected that hesperidin mixed with CT could produce a better weight-control effect.

CT was characterized by Fourier transform infrared spectroscopy (FTIR) and nuclear magnetic resonance (NMR) spectroscopy to identify functional groups and the synthetic material structure. WST-1 assay and live/dead staining were used to determine cell viability and biocompatibility. The mucoadhesive property was verified using u-slides seeded with IEC-6 cells and FITC-labeled CT (CTF). The obesity model was developed by C57BL/6 mice fed a high-fat diet. Finally, tissue H&E staining, complete blood count, and serum biochemical analysis were used to evaluate the impact of daily feeding materials on the animal body or tissue.

## 2. Materials and Methods

### 2.1. Synthesis of Chitosan-TGA (CT)

To synthesize the thiolated chitosan, 1 g of chitosan powder was mixed into 50 mL of 0.1 M acetic acid solution, and 0.37 mL of thioglycolic acid (TGA) was added and the mix stirred evenly. Then, 0.098 mL of BDDE was dissolved in 50 mL of isopropanol and mixed into the solution mentioned above. After stirring for three hours in the dark at room temperature, the solution was dialyzed in ddH2O using a dialysis membrane (MWCO: 12,000 Da) for 72 h. The solution was further lyophilized and stored at room temperature for the following experiments.

### 2.2. FTIR Spectroscopy Analysis

The FTIR was used for the determination of functional groups within the candidate materials. A total reflection FTIR spotlight 200i (Perkin Elmer, Waltham, MA, USA) was used to conduct the measurements. The sample was scanned 16 times with a resolution of 4 cm^−1^. The sample was analyzed by transmittance mode in the range of 500–4000 cm^−1^. Absorption peaks of each functional group were analyzed.

### 2.3. NMR Spectroscopy Analysis

An amount of 8 mg chitosan or CT was dissolved in 1 mL of 0.1 M CD3COOD/D2O solution and filtered with a 0.22 μm filter. The inspection was performed by the National Taiwan University Precious Instrument Center. The 1H-NMR was measured with a 500 MHz superconducting magnet on a Bruker Avance III 500 spectrometer.

### 2.4. Cytotoxicity of CT

The cytotoxicity of the CT was evaluated by WST-1 assay and live/dead staining. L929 cells were seeded into the 96 wells, at a cell density of 7000 per well. CT was dissolved in 0.1 M acetic acid aqueous solution at a concentration of 3 mg/mL and mixed with a medium in a ratio of 1:5. The positive control was 0.2 g/mL ZDEC extract, while the negative control was 0.2 g/mL Al_2_O_3_ extract. After co-culturing the cells with the extracts for 24 h, the WST-1 assay was added for 1.5 h of reaction. Finally, an ELISA reader was used to detect the absorbance wavelength at 450 nm to measure cell activity. Live/dead staining was used to evaluate cytotoxicity. The L929 cell line was first seeded in a 24-well plate at a density of 20,000 cells/well. After one day of incubation, 0.5 mL of 1.5% chitosan or CT was added in a serum-free MEM medium to each well. After another 24 h of incubation, the cell culture medium was removed, and the cells were thoroughly washed by phosphate-buffered saline (PBS) 3 times. Triton X-100, as a positive control, was added 5 min before staining. Then, a staining solution containing calcein-AM (2 μM) and ethidium homodimer-1 (EthD-1) (4 μM) was added and allowed to react in the wells for 30 min. After washing with PBS, the cells were observed using a fluorescence microscope to evaluate the cytotoxicity of the chitosan and CT.

### 2.5. In Vitro Mucus Adhesion Test

The mucus adhesion test was adopted to gauge the enhanced mucoadhesive properties of CT. IEC-6 cells. Intestine epithelial cells, which could secrete mucosa layers to mimic the microenvironment of the gastrointestinal tract were utilized. IEC-6 was seeded in the µ-Slides (from ibidi µ-Slide I 0.8 Luer) at a cell density of 400,000 cells/mL overnight. The cells were first stained with Hoechst 33,342 (1 μg/mL) within the medium for 15 min. Then, 6 mg/mL of chitosan-FITC (CF) or CT-FITC (CTF) in 0.1 M acetic acid solution was diluted with the medium at a ratio of 1:1 and incubated with IEC-6 on the µ-slides for 1 h. The microfluidic device generated a constant pumping of culture medium into the slides at a flow rate of 3 mL/h. Finally, at the times of 0, 1, and 2 h, a fluorescent microscope was used to observe the fluorescence intensity of the materials bonded with the cells to evaluate the adhesion of the materials.

### 2.6. Oral Glucose Tolerance Test (OGTT) and Intraperitoneal Glucose Tolerance Test (IPGTT)

Eight-week-old C57BL/6 male mice were starved for 18 h and 6 mice were placed in each group including the control group, the positive control group, and the CT group. Except for the control group, positive control, and CT groups mice were gavaged with PBS or CT (250 mg/kg) respectively 1 h before oral administration of glucose solution (3 g/kg mice) in OGTT. The IPGTT, positive control, and CT groups were gavaged with PBS or CT (250 mg/kg) respectively 1 h before intraperitoneal injections of glucose solution (3 g/kg mice). Mice without glucose gavage served as the control group. Glucose levels were measured using a glucose meter (Accu-Chek Instant, Roche, Basel, Switzerland) every 30 min for 120 min. Moreover, the incremental area under the curve (iAUC) was used to diagnose the response of OGTT.

### 2.7. In Vivo Adhesion Test of CT

The adhesion properties of CF or CTF in vivo were evaluated by the IVIS Luminar II in vivo imaging system (Perkin Elmer, Waltham, MA, USA). C57BL/6 male mice were gavaged with CF or CTF with a dose of 250 mg/kg mice. The gastrointestinal tracts were harvested at each time point of 1, 2, 4, and 24 h.

### 2.8. In Vivo Study

C57BL/6 (4-week-old) male mice were used to test the long-term treatment of CT or CTH. The operations following lasted eight weeks. The mice in the control group were fed with a normal diet (ND) and were gavaged with phosphate-buffered saline (PBS) daily. The mice in the high-fat diet (HFD) group were fed a 60 percent high-fat diet from Dyets (DYET# 112252) and gavaged with PBS. The mice in the HFD + CT group or HFD + CTH group were fed with the same 60 percent high-fat diet but gavaged with different material experiment solutions (250 mg/kg) daily. Every week, the body weight and food intake were measured. A full-body scan employing micro computerized tomography (SKYSCAN, Bruker, Billerica, MA, United States) with a resolution of 35 µm was done at the end of the in vivo investigation to examine the effects of material gavaging on adipose tissue distribution.

### 2.9. Statistics

The results with at least three replicates were presented as “mean ± standard deviation.” To assess the statistical significance of the experiment, a one-way ANOVA with multiple comparison tests was used. At a *p*-value of less than 0.05, differences were judged significant *p* < 0.05, *; *p* < 0.01, **; *p* < 0.001, ***.

## 3. Results

### 3.1. Material Molecular Structure Analysis

**Figure 1:**
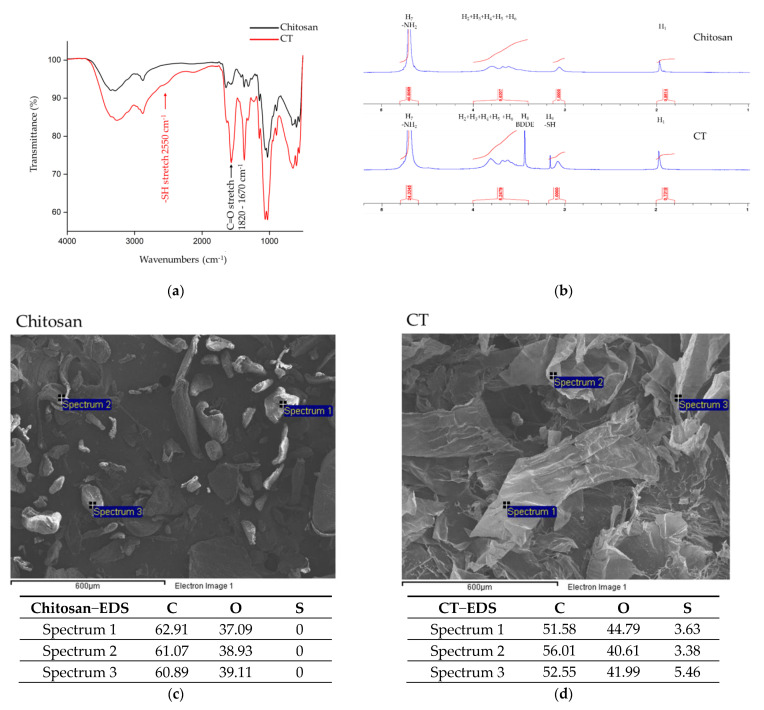
Material molecular structure analysis of CT. (**a**) The FTIR result of chitosan and CT. (**b**) ^1^Hydrogen NMR spectra confirmed the structure of chitosan and CT. EDS results showed that there were sulfur signals on the surface of CT (**d**) but not on chitosan (**c**).

FTIR, which could measure each functional group’s own discrete vibrational energy, was used to identify the functional groups of chitosan and CT (Figure 1a). Both chitosan and CT exhibited similar characteristics of OH absorption at 3600–3200 cm^−1^ and CH stretching at 3000–2850 cm^−1^. As our expectation, there was an absorption band for the thiol group at 2550 cm^−1^ for CT. Apparently, these results indicated that the amino groups in chitosan were successfully modified with thiol groups. By analyzing the 1H-NMR spectrum, the structure of CT could be verified. As seen in Figure 1b for chitosan, H1 appeared at 2.0 ppm, H2, H3, H4, H5, H6 were distributed between 3.4 ppm and 3.8 ppm, which was comparable to the NMR spectroscopy of chitosan in previous studies [23]. In the 1H-NMR spectrum of CT, BDDE, the cross-linking agent, was detected at 3.4 ppm. CT also exhibited an obvious characteristic peak at 3.2 ppm, confirming that a thiol group was grafted onto the molecule (Figure 1b). In the EDS analysis, sulfur signals were detected on the CT surface (Figure 1c). The collaborating results confirmed that CT was successfully synthesized by conjugating TGA with chitosan by BDDE crosslinker.

### 3.2. Cytotoxicity and Mucus Adhesion Test of CT In Vitro

**Figure 2:**
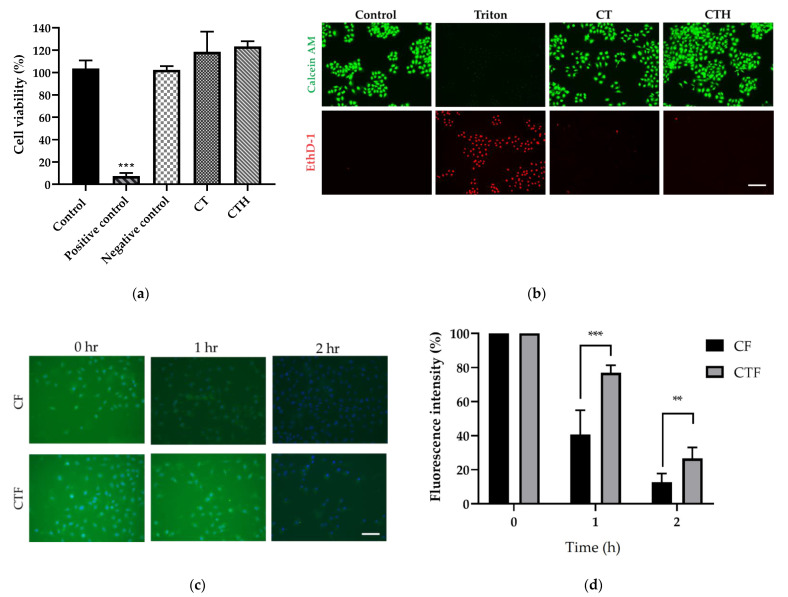
Evaluation of biocompatibility and mucoadhesion of CT. (**a**) WST-1 test to evaluate cell viability of CT and CTH with L929 cell. (*n* = 6, *** *p* < 0.001) (**b**) Cytotoxicity of CT and CTH tested using live/dead staining. Living cells were stained with calcein AM and dead cells with homodimer-1 (EthD-1) (scale bar = 100 μm). (**c**) In vitro adhesion evaluation of chitosan -FITC (CF) and CT-FITC (CTF). The fluorescence microscope was used to observe material adhesion on mucus. (**d**) Fluorescence quantification of the in vitro adhesion test. One-way ANOVA (*n* = 3, ** *p* < 0.01 *** *p* < 0.001).

Following the specifications of ISO10993, the cytotoxicity of CT was evaluated by the WST-1 cell viability assay and live/dead staining. To determine the cytotoxicity of chemicals in vitro, the WST-1 assay, a tetrazolium salt, which is converted into a colored dye by mitochondrial dehydrogenase enzymes, is the most commonly used test. The control group consisted of conventional culture media; the positive control consisted of ZDEC extract, and the negative control group consisted of Al_2_O_3_ extract. The experimental group comprised chitosan or CT dissolved in the medium. Results showed that the materials were biocompatible and had negligible toxicity in L929 cells (Figure 2a). Live/Dead assay was performed to evaluate whether the material would showcase obvious cytotoxicity toward the L929 cell line as shown in (Figure 2b). The cell-permeant dye calcein AM can be used to determine the viability of most eukaryotic cells. Ethidium homodimer 1 dye (EthD-1) is highly positively charged. Therefore, it can be used to detect nucleic acids in solutions or to stain dead cells selectively. In both the chitosan and CT group, cell morphology was not significantly altered, and most of the cells were alive. Consequently, the above results indicated that chitosan and CT did not exhibit cytotoxicity and possessed desirable biocompatibility.

To test the mucoadhesive properties of the thiolated material, a µ-slide containing IEC-6 cells (small intestine epithelial cell) was used, followed by a constant flow of medium. For clear observation, nuclei were stained with Hoechst 33342, and candidate materials were labeled with FITC, demonstrated in blue fluorescence for nucleus and green fluorescence for the material respectively. At the beginning of the test, most of the materials were presented in the µ-slide, causing a strong background and obvious overlapping of signals from nuclei and materials. After 2 h of medium washing, a portion of the materials was flushed away, resulting in a decrease in green fluorescence. Compared with CF, CTF had a stronger signal remaining, indicating the ability to extend its retention time on the mucus with the thiolated modification (Figure 2c). Utilizing ImageJ software, the FITC fluorescence intensity was calculated to quantitate the remaining materials on the mucus, where the initial fluorescence intensity (0 h) was normalized to 100%. As shown in Figure 2d, the signal of CF was reduced to 40%, whereas the signal of CTF only decreased to 77%, verifying the efficacy of thiolation.

### 3.3. Functional Barrier Test and Mucosal Adhesion In Vivo

**Figure 3:**
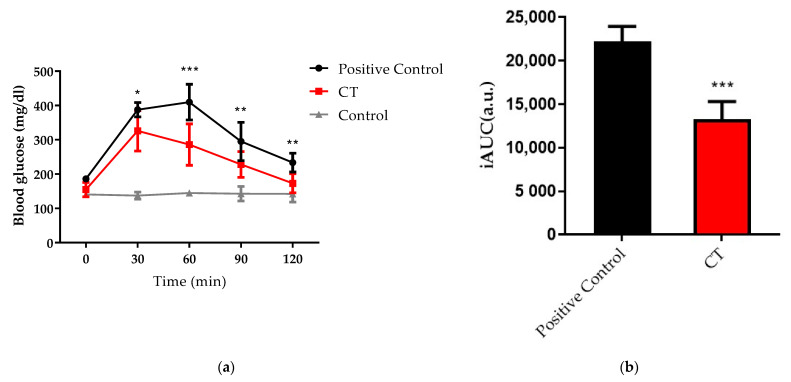

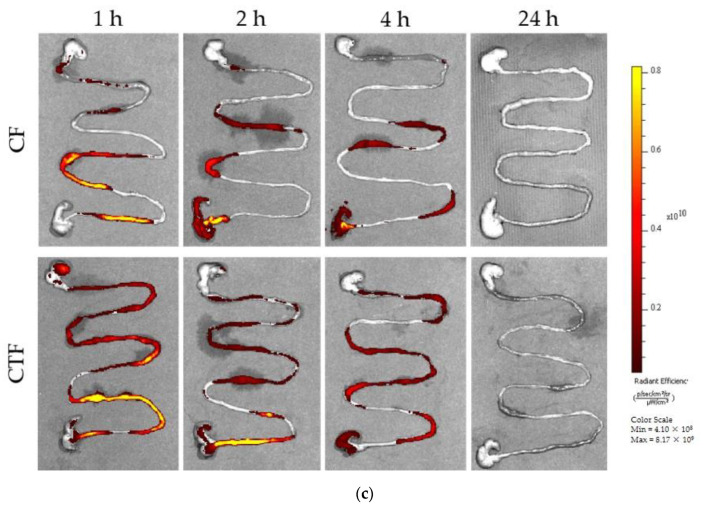
Oral glucose tolerance and in vivo adhesion evaluation. (**a**) Blood glucose response post-CT gavage (250 mg/kg). Two-way ANOVA with multiple comparisons (*n* = 6, * *p* < 0.1 ** *p* < 0.01 *** *p* < 0.001). (**b**) iAUC of the OGTT curves. One-way ANOVA with multiple comparisons (*n* = 6, * *p* < 0.1 ** *p* < 0.01 *** *p* < 0.001 compared to positive control) (**c**) Gastrointestinal tracts of mice were harvested from the stomach to cecum after gavage with CF or CTF (250 mg/kg) at each time point followed by IVIS in vivo imaging systems.

An in vivo investigation of the therapeutic effects of CT on intestinal absorption was necessary. In addition, as obesity could impair glucose tolerance and insulin function [24], an oral glucose tolerance test (OGTT) was performed to determine whether the materials would block glucose absorption in the intestine after forming a physical barrier. C57BL/6 male mice (8 weeks old) were first gavaged with CT to form an intestinal coating. Afterwards, glucose solutions were administered to each group orally except for the control group. Judging from the glucose response curves, the CT group had a much lower level of blood glucose at 326 mg/dL than the positive control group (387 mg/dL) after 30 min. In both positive control and the CT group, blood sugar levels returned to their baseline levels after 120 min due to the action of insulin (Figure 3a). As for quantification, iAUC was significantly reduced in the CT group by 40.27% (Figure 3b), indicating that CT indeed formed a barrier coating on the small intestine to inhibit glucose absorption.

The adhesion properties of CF and CTF on the small intestinal mucosa were verified in C57BL/6 male mice. The gastrointestinal tracts were harvested after fluorescence material gavaging at 1, 2, 4, and 24 h respectively. Using an IVIS imaging system, the fluorescence signal was detected in the gastrointestinal tract from the stomach to the cecum. The signals of fluorescence intensity quickly began to differ between CF and CTF after 1 h. The signal of CF deteriorated notably over the course of four hours, CTF, while on the other hand, it still had strong fluorescent signals (Figure 3c). After 24 h, neither CF nor CTF showed any fluorescence. Accordingly, we assumed that the material would not adhere permanently to the gastrointestinal tract. Compared with the CF, CTF formed better adhesion to the intestinal tract of mice in fluorescence imaging, as indicated by an in vitro adhesion test.

### 3.4. CT as a Drug Release Hydrogel with a Long-Term Effect in an HFD Model

There were four groups in the long-term evaluation of materials: the normal diet group (ND), the high-fat diet group (HFD), and the high-fat diet plus CT group (HFD + CT). In addition, hesperidin is a flavanone glycoside that has been known to be effective in relieving symptoms such as hyperlipidemia and hyperglycemia [21]; therefore, a high-fat diet plus a hesperidin-loaded CT group (HFD + CTH) was added to evaluate whether CT could be used as an anti-obesity drug delivery system. PBS was given to the control group as well as the group with HFD for eight weeks. A daily dose of 250 mg/kg mice of material was given to both the CT and CTH groups for eight weeks. During the experiment, the bodyweight of mice was measured once a week, and the rate at which their weight changed was recorded.

**Figure 4:**
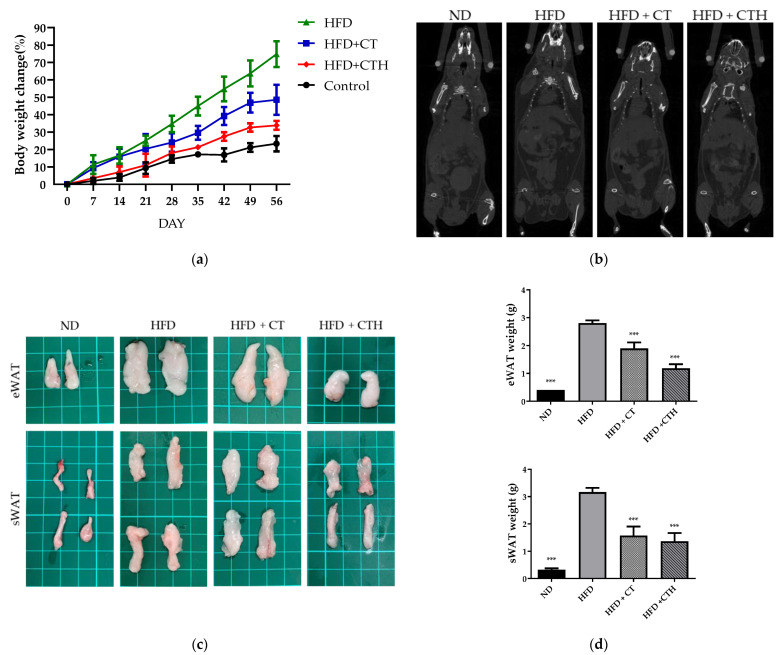
Evaluation of the bodyweight and fat accumulation (**a**) Eight-week weight record for normal diet group (ND), high-fat diet group (HFD), high-fat diet with CT group (HFD + CT), and high-fat diet with CT combined hesperidin (HFD + CTH). Two-way ANOVA with multiple comparisons (*n* = 6, *** *p* < 0.001 for HFD + CT vs. HFD). (**b**) CT images of mice with a resolution voxel spacing of 35 μm. (**c**) Representative images of epididymal white adipose tissue (eWAT) and subcutaneous white adipose tissue (sWAT). (**d**) Weight of eWAT and sWAT. One-way ANOVA with multiple comparisons compared with HFD (*n* = 6 for each group, *** *p* < 0.001).

In comparison to the HFD group, the weight gains of the CT group and CTH group were significantly lower after 35 days of treatment (Figure 4a), with the CTH group showing slightly better results than the CT group. The difference in weight changes between each group could also be observed clearly in Figure S1. Compared with the HFD group, the HFD + CT group had a significant reduction in weight gain of 26.28% and the HFD + CTH group had a greater reduction of 40.91%. Computed tomography (CT) scan, a non-invasive medical imaging technique, was used to visualize the accumulation of adipose tissue. In both the CT and CTH groups, there was a significant decrease in adipose distribution compared to the HFD group (Figure 4b).

In the following eight-week period, animals were sacrificed, and the epididymal white adipose tissue (eWAT) and subcutaneous white adipose tissue (sWAT) were weighed (Figure 4c). The HFD group, HFD + CT, and the HFD + CTH group showcased a significant reduction in adipose tissue as compared to the HFD group after dissection. eWAT was 50.15 percent lower in the HFD + CT group than in the HFD group, whereas eWAT of the HFD + CTH group was 56.8 percent lower than in the HFD group (Figure 4d). As shown in Figure 4d, the sWATs of groups HFD + CT and HFD + CTH were significantly lower than that of the HFD group by 32.14 percent and 57.85 percent. The results above indicate that both the CT and CTH groups could successfully reduce eWAT and sWAT; However, CTH was proved to be more successful in reducing eWAT.

### 3.5. The Result of H&E Staining

**Figure 5:**
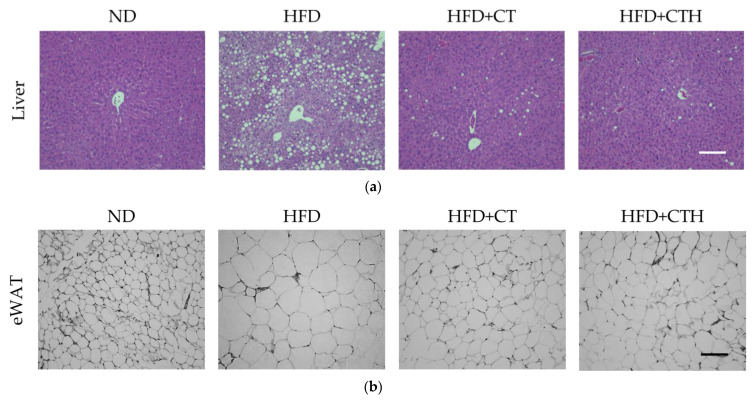
Results of hematoxylin and eosin stain. HE staining for (**a**) liver and (**b**) epididymal white adipose tissue (eWAT) (scale bar = 100 μm).

A large amount of lipid was accumulated in the liver of the HFD group, whereas the control group had much less lipid accumulation in liver H&E staining despite HFD, CT, and CTH having significantly reduced fatty liver symptoms, according to the results in Figure 5a. Furthermore, no differences were found in the H&E staining of the stomach or the small intestines (Figure S2). The H&E staining results of eWAT (Figure 5b) showed that the adipocyte diameters and volumes in the HFD group were larger than the control, as well as the HFD + CT, and HFD + CTH groups. It is speculated that CT or CTH suppresses nutrient absorption, thereby slowing lipid synthesis.

### 3.6. Serum Biochemical Analysis and Complete Blood Count

C57b/6 mice were sacrificed by carbon dioxide inhalation, and blood samples were taken by cardiac puncture using a 26G needle for the biochemical analysis of serum and complete blood count. In the serum biochemical analysis, aspartate aminotransferase (AST) and alanine aminotransferase (ALT) were widely used as enzyme biomarkers of liver injury. Furthermore, biochemical markers associated with lipid metabolisms, such as the total cholesterol (TC), triglycerides (TG), high-density lipoproteins (HDL), and low-density lipoproteins (LDL), were also investigated. Finally, kidney dysfunction can be evaluated by testing blood urea nitrogen (BUN) and creatinine (Cre) in serum biochemical analysis (Table 1).

The results of the complete blood count (CBC) including white blood cells (WBC), neutrophil (NEUT), eosinophil (EO), basophil (BASO), monocyte (MO), lymphocyte (LYMPH), red blood cell (RBC), hemoglobin (HGB), hematocrit (HCT), mean corpuscular volume (MCV), mean corpuscular hemoglobin (MCH), reticulocyte count (RET), mean corpuscular hemoglobin concentration (MCHC), and platelets (PLT) are within the normal range compared with the control group (Table 2). According to the CBC results, the gavaged CT or CT with hesperidin will not cause adverse immune reactions in the body, making the materials safe for experimental use in animals.

## 4. Discussion

Obesity is defined as the presence of excessive body fat that poses a health risk, including an increased risk of chronic diseases like cancer [25,26]. According to estimates, these preventable diseases will result in a combined medical costs of $48–66 billion/year in the US and £1.9–2 billion/year in the UK by 2030 [27]. Natural materials such as chitosan are gradually being developed, modified, and tested due to the increase in overweight or obese people in the world. In this study, we used chitosan as main material and BDDE as a crosslinking agent and combined it with thioglycolic acid as a CT. BDDE is a bifunctional compound containing epoxy groups on both ends that can be used to crosslink nucleophilic molecules such as hydroxyl group, amino group, and sulfhydryl group through a ring-opening mechanism. Ellman’s assay result also revealed sulfhydryl groups on CT (Figure S3). Our research examined the possibility that chitosan hydroxyls and amines could form ether bonds with free thiols on TGA. In combination with the above results, CT was confirmed by functional group analysis (Figure 1a,b) and the sulfur signal on the surface was determined in the EDS result (Figure 1c,d).

Intestines contained a lot of mucosa of epithelium and connective tissue. The epithelial and mucosal tissue, which provided the first defense system, contained redox pairs, such as glutathione, cysteine, and thioredoxin reductase [28]. Due to these redox pairs, the mucosa contained enough thiol groups to ensure optimal adhesion between mucoadhesive materials and the mucosa. Moreover, the mucoadhesive material in this study will be excreted, thus eliminating potential toxicity concerns or the risk of accumulating bioactive molecules. From in vivo verification (Figure 3), the results demonstrate that there is a good glucose blocking effect after CT gavaging. In vitro glucose barrier functional tests have also established similar results (Figure S4). However, in the IPGTT, the glucose response did not differ, suggesting that the decreased glucose response is due to a localized barrier coating instead of a systemic effect (Figure S5). It is also evident from the results of IVIS that CT can increase the time of stay in the gastrointestinal tract but will not accumulate permanently.

Hesperidin has anti-inflammatory, anti-oxidative, and anti-cancer properties that contribute to lowering cholesterol levels, blood pressure, and obesity [29]. To be more specific, hesperidin modulates AMPK and PPAR signaling pathways in obesity treatment, which affects the antioxidant index, anti-apoptosis, and NF-κB signaling pathways indirectly [30]. CT added with hesperidin (CTH) not only had the effect of CT blocking gastrointestinal absorption, with the effect of hesperidin, it could also make further improvements in terms of weight loss. However, there have been many strategies proposed to help people lose weight and maintain their weight, including caffeine, ephedra, capsaicin, and green tea, since they have the potential to increase energy expenditure and counter the decline in metabolic rate associated with weight increase [31]. In the future, we will try adding auxiliary additives to CT materials to assist their effectiveness

## 5. Conclusions

In this study, we crosslinked chitosan with BDDE and grafted TGA onto the chitosan, creating chitosan-TGA (CT). The mucoadhesive CT had better mucoadhesive properties than unmodified chitosan. The prepared hesperidin-containing CT (CTH) and CT had no cytotoxicity in vitro and no systemic toxicity in vivo.

At the same time, CT could be excreted from the body after 24 h of feeding materials and will not accumulate in the body. Hesperidin mixed with CT (CTH) not only effectively reduced the body weight by 40.91% compared with the HFD group, but also greatly reduced the accumulation of body fat. From the results, it could be shown that the developed CTH could be used as a biosafety mucosal gel for obesity treatment. CT as a barrier to inhibit absorption can also be used as a drug carrier for different natural Chinese herbal medicines to treat obesity or nonalcoholic fatty liver disease (NAFLD) in the future.

## Figures and Tables

**Table 1:** Serum biochemical assessment.

	ND	HFD	HFD + CT	HFD + CTH
TC (mg/dL)	89.40 ± 12.41	158.60 ± 8.46 ***	145.80 ± 19.63 ***	144.10 ± 9.31 ***
TG (mg/dL)	93.32 ± 26.73	128.20 ± 21.83 *	116.60 ± 13.57 *	105.3 ± 7.55
HDL (mg/dL)	75.6 ± 14.52	140.90 ± 7.89 ***	123.80 ± 14.61 ***	122.70 ± 8.59 ***
LDL (mg/dL)	8.63 ± 3.93	15.84 ± 2.20 ***	13.96 ± 2.91 **	13.93 ± 2.29 **
AST (U/L)	108.1 ± 26.14	286.3 ± 91.52 ***	153.4 ± 51.6 ^###^	88.24 ± 28.7 ^###^
ALT (U/L)	35.62 ± 6.37	40.63 ± 8.70	37.64 ± 8.26	33.73 ± 11.73
BUN (mg/dL)	20.86 ± 3.91	19.48 ± 2.61	18.35 ± 2.57	17.97 ± 2.33
Crea (mg/dL)	0.25 ± 0.05	0.23 ± 0.05	0.21 ± 0.03	0.23 ± 0.05

One-way ANOVA with multiple comparisons (*n* = 6 for each group, * *p* < 0.05, ** *p* < 0.01, *** *p* < 0.005 compared to ND, ### *p* < 0.005 compared to HFD).

**Table 2:** Complete blood count.

	ND	HFD	HFD + CT	HFD + CTH
RBC (M/μL)	10.25 ± 0.62	9.975 ± 0.261	10.71 ± 0.213	10.48 ± 0.376
HGB (g/dL)	14.57 ± 0.85	14.25 ± 0.212	15.41 ± 0.3586	15.10 ± 0.916
HCT (%)	55.71 ± 3.75	53.05 ± 1.061	53.62 ± 1.45	54.50 ± 3.830
MCV (fL)	52.98 ± 1.033	53.20 ± 0.282	50.06 ± 1.276	51.93 ± 1.790
MCH (pg)	14.62 ± 0.25	14.30 ± 0.141	14.39 ± 0.209	14.40 ± 0.360
MCHC (g/dL)	27.61 ± 0.516	26.90 ± 0.141	28.77 ± 0.9447	27.70 ± 0.264
RET (K/μL)	429.4 ± 106.8	514.8 ± 18.24	490.3 ± 79.54	502.8 ± 113.9
PLT (K/μL)	1124 ± 259.0	802.0 ± 114.6	1258 ± 140.30	928.7 ± 328.7
WBC (K/μL)	4.631 ± 2.186	3.650 ± 0.480	6.193 ± 1.229	2.547 ± 0.140
NEUT (K/μL)	1.181 ± 1.015	0.755 ± 0.304	1.324 ± 0.57	0.450 ± 0.155
LYMPH (K/μL)	3.263 ± 1.568	2.570 ± 0.08485	7.523 ± 2.474	1.887 ± 0.2228
MONO (K/μL)	0.1589 ± 0.1776	0.190 ± 0.084	0.257 ± 0.150	0.1300 ± 0.079
EO (K/μL)	0.026 ± 0.018	0.1267 ± 0.006	0.233 ± 0.105	0.076 ± 0.035

## Supplementary Materials

The following are available online at https://www.mdpi.com/article/10.3390/nu13124405/s1, Figure S1: Representative images of the control group, the HFD group, HFD + CT group and the HFD + CTH group, Figure S2: H&E staining of stomach and small intestine, Figure S3: Ellman’s assay of CT, Figure S4: The glucose barrier functional test, and Figure S5: IPGTT.
