# Supplementary material for: Thiolated Chitosan as an Intestinal Absorption Carrier with Hesperidin Encapsulation for Obesity Treatment
Source: Nutrients. 2021 Dec 9;13(12):4405. doi: 10.3390/nu13124405 (PMC8706427; doi:10.3390/nu13124405)
Supplement: Supplementary file 1 [file nutrients-13-04405-s001.zip › nutrients-1449379-supplementary.pdf]

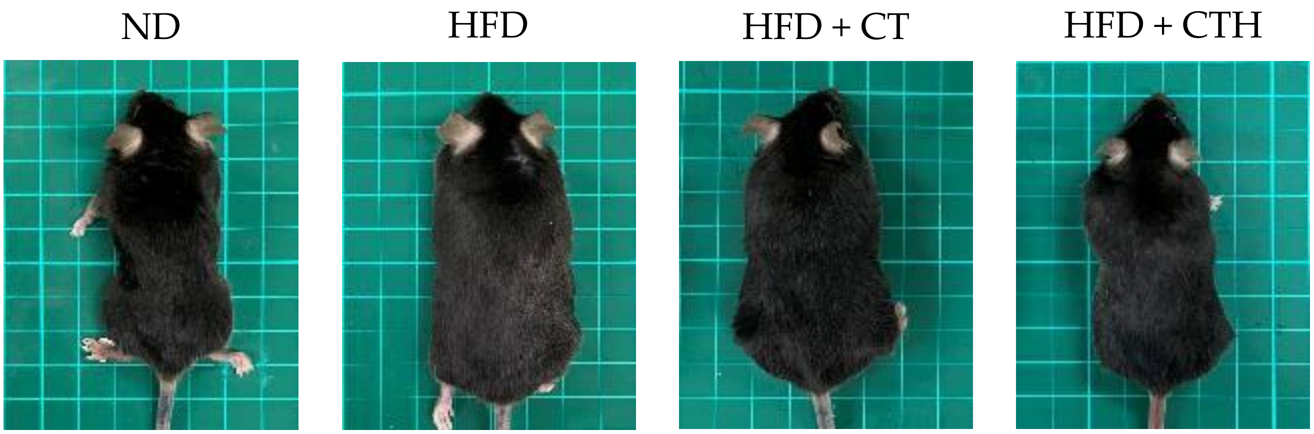

Figure S1. Representative images of the control group, the HFD group, HFD + CT group and the HFD + CTH group

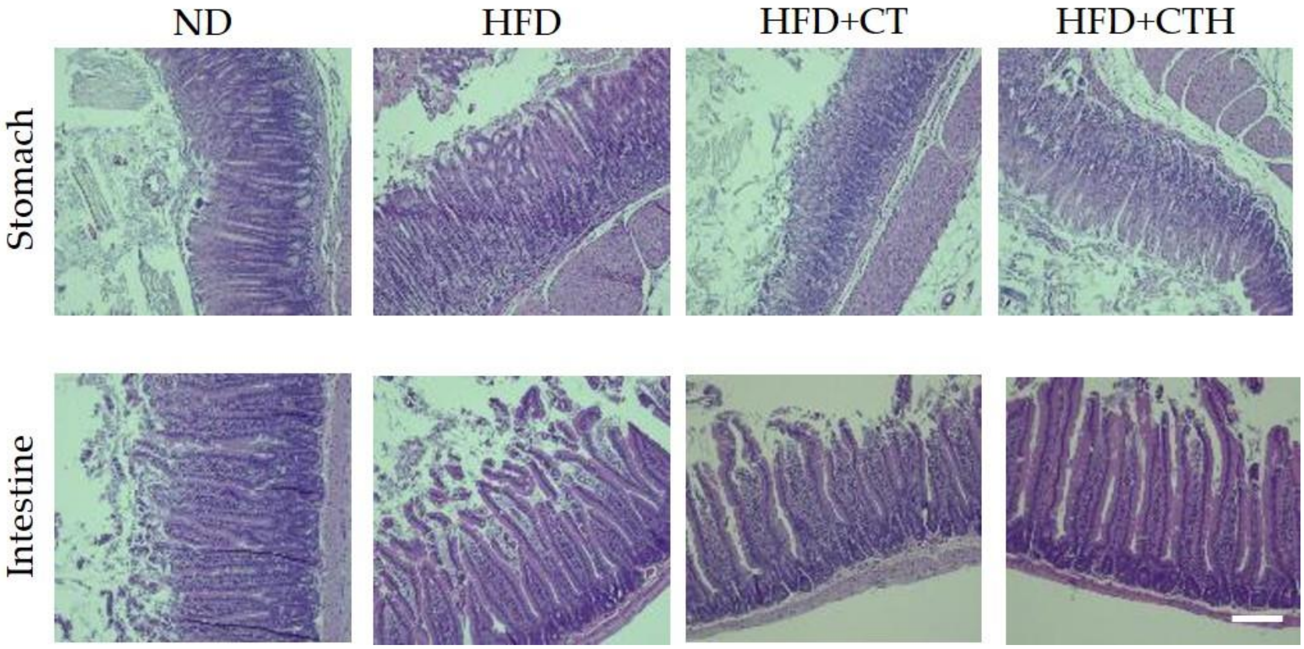

Figure S2. H&E staining of stomach and small intestine.
(scale bar = 100 μm)

	OD	Concentration (mM)
Chitosan-TGA (2 mg/mL)	1.6048	0.8583

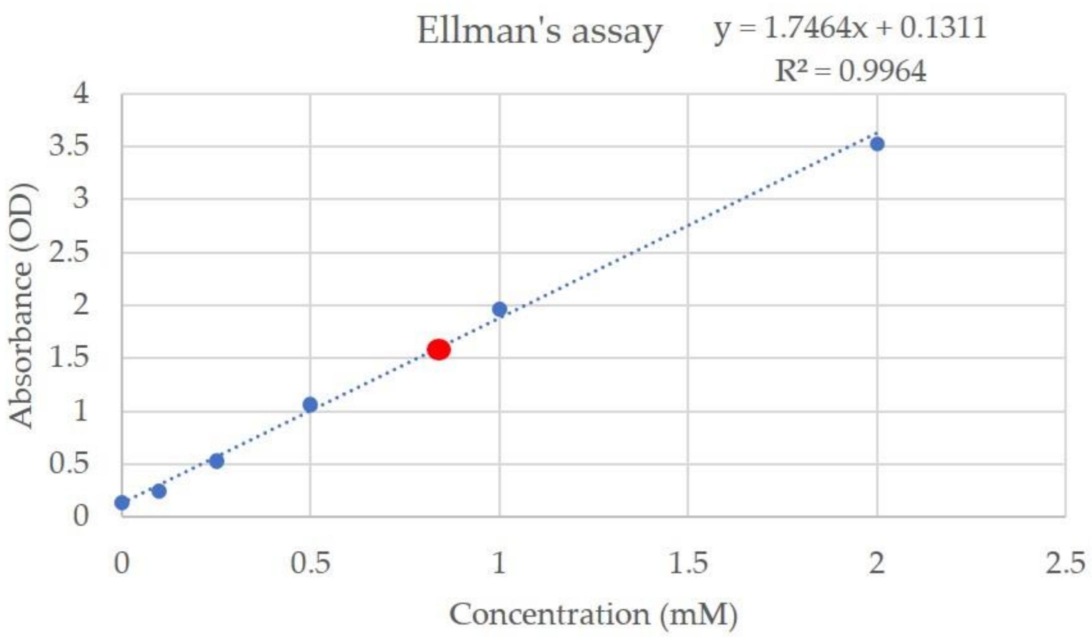

Figure S3. Ellman's assay of CT

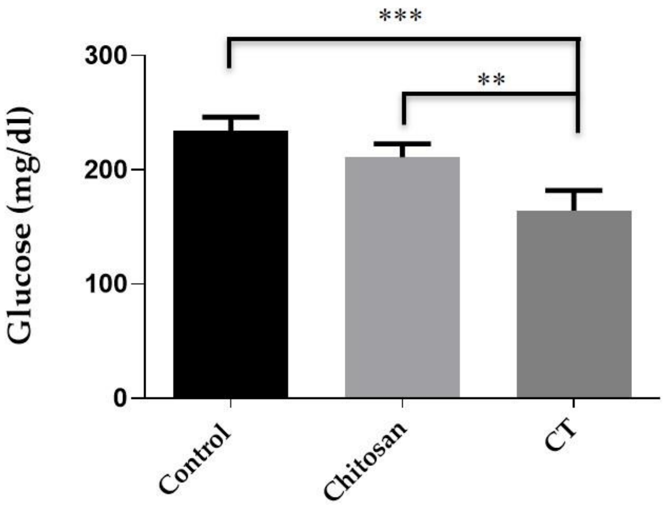

Figure S4. The glucose barrier functional test
n = 6 for each group, **P < 0.01,***P <0.005

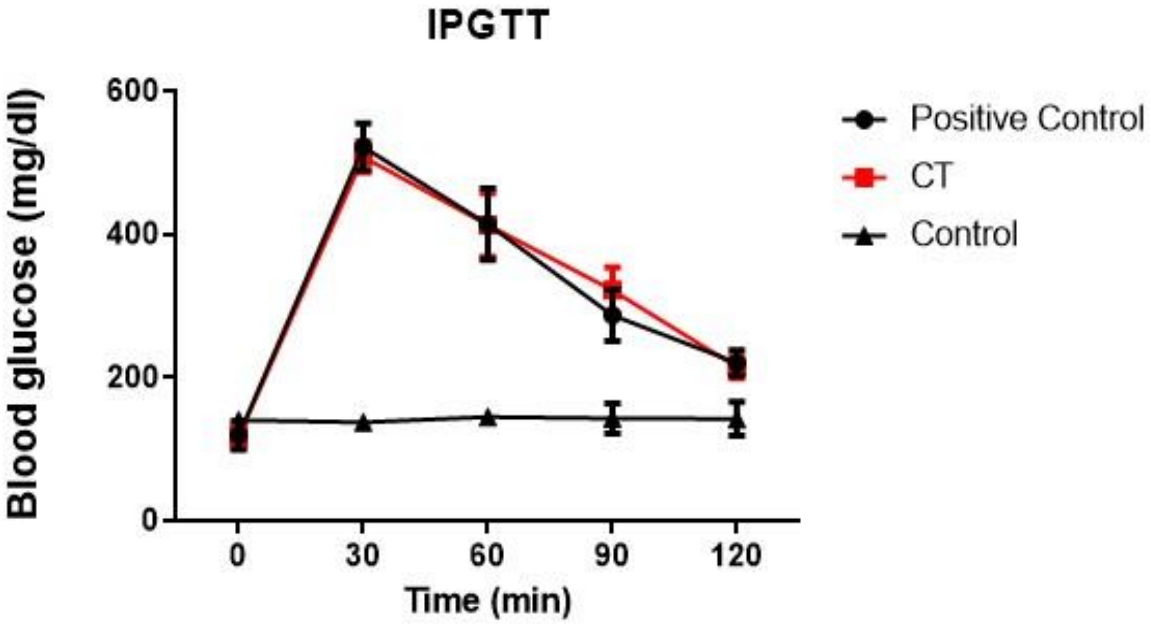

Figure S5. IPGTT
n = 3 for each group
